# Supplementary material for: An ambiguous N-terminus drives the dual targeting of an antioxidant protein Thioredoxin peroxidase (TgTPx1/2) to endosymbiotic organelles in Toxoplasma gondii
Source: PeerJ. 2019 Jul 18;7:e7215. doi: 10.7717/peerj.7215 (PMC6642795; doi:10.7717/peerj.7215)
Supplement: Supplemental Information 1 — Here, ACP is used as an apicoplast marker protein while SP-SOD2-GFP and MitoTracker Red are used as mitochondrial markers (Mito). [file peerj-07-7215-s001.docx]

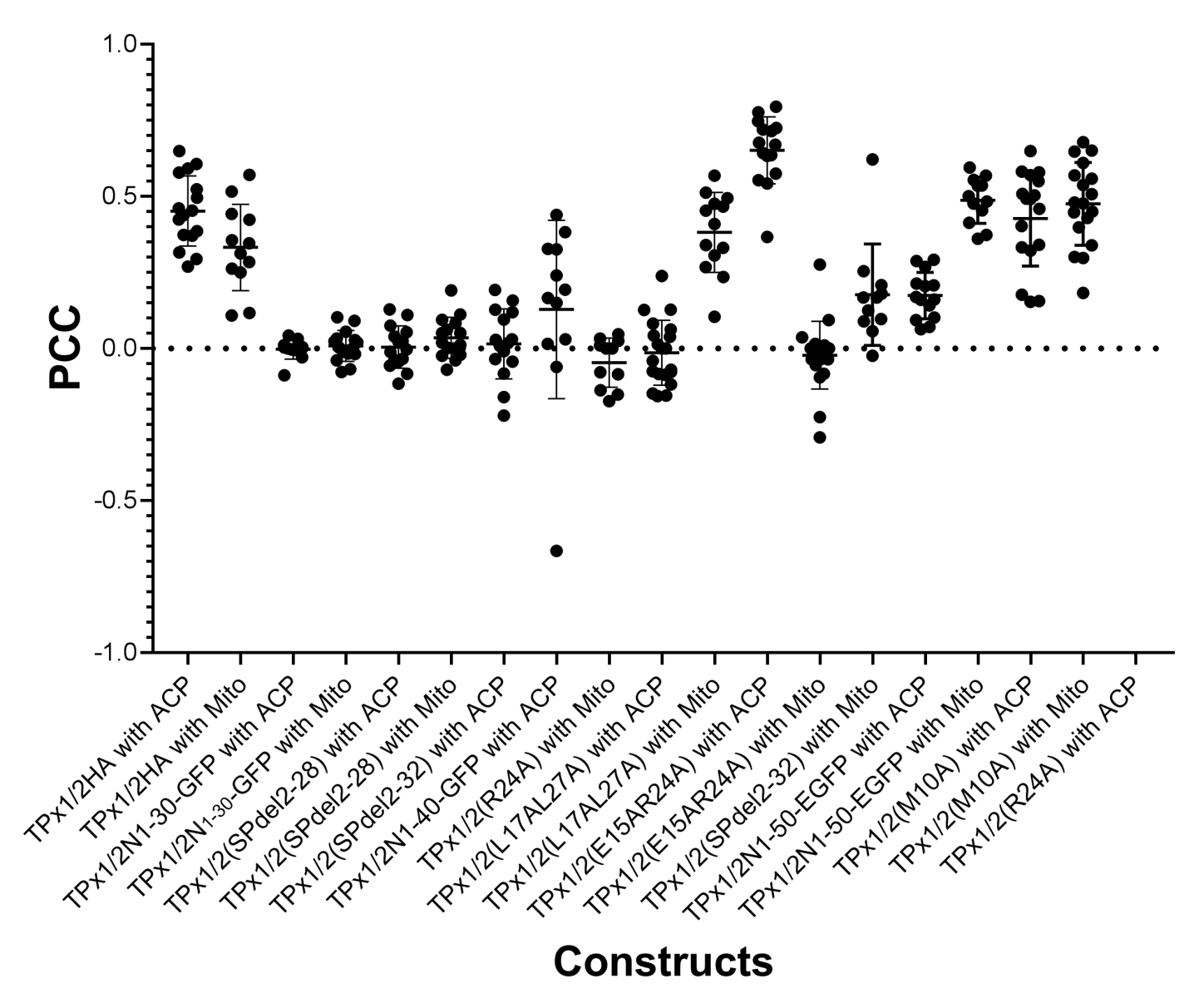


**Supplementary figure S1**: A dot plot of the Pearson’s correlation coefficients (PCC) for co‑localization between the proteins expressed by the individual constructs and the appropriate markers used in this study. Here, ACP is used as an apicoplast marker protein while SP‑TP‑SOD2-GFP and MitoTracker Red are used as mitochondrial markers (Mito).
