# Supplementary material for: An ambiguous N-terminus drives the dual targeting of an antioxidant protein Thioredoxin peroxidase (TgTPx1/2) to endosymbiotic organelles in Toxoplasma gondii
Source: PeerJ. 2019 Jul 18;7:e7215. doi: 10.7717/peerj.7215 (PMC6642795; doi:10.7717/peerj.7215)
Supplement: Supplemental Information 2 [file peerj-07-7215-s002.docx]

**
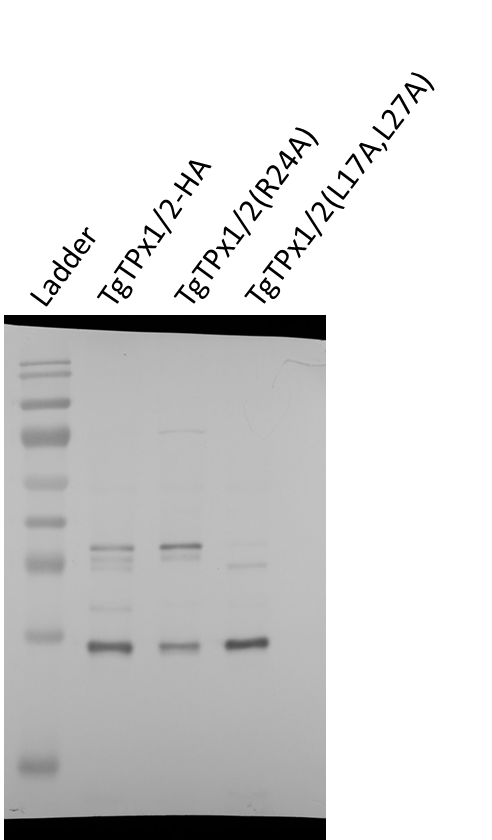
**

**S****upplementary figure S2**: **Full-length Western blot displayed in main figures**. High contrast images were not generated and hence multiple exposure images are not included here.
