# Supplementary material for: An ambiguous N-terminus drives the dual targeting of an antioxidant protein Thioredoxin peroxidase (TgTPx1/2) to endosymbiotic organelles in Toxoplasma gondii
Source: PeerJ. 2019 Jul 18;7:e7215. doi: 10.7717/peerj.7215 (PMC6642795; doi:10.7717/peerj.7215)
Supplement: Supplemental Information 3 — α-helix predictions for the N-terminus of (A) apicoplast targeted protein TgACP and (B) mitochondrial protein HSP60. The blue arrows mark the hydrophobic residues while the red arrows label the positively charged amino acids on the helical projections for the N-terminus of the proteins used for analysis. [file peerj-07-7215-s003.docx]

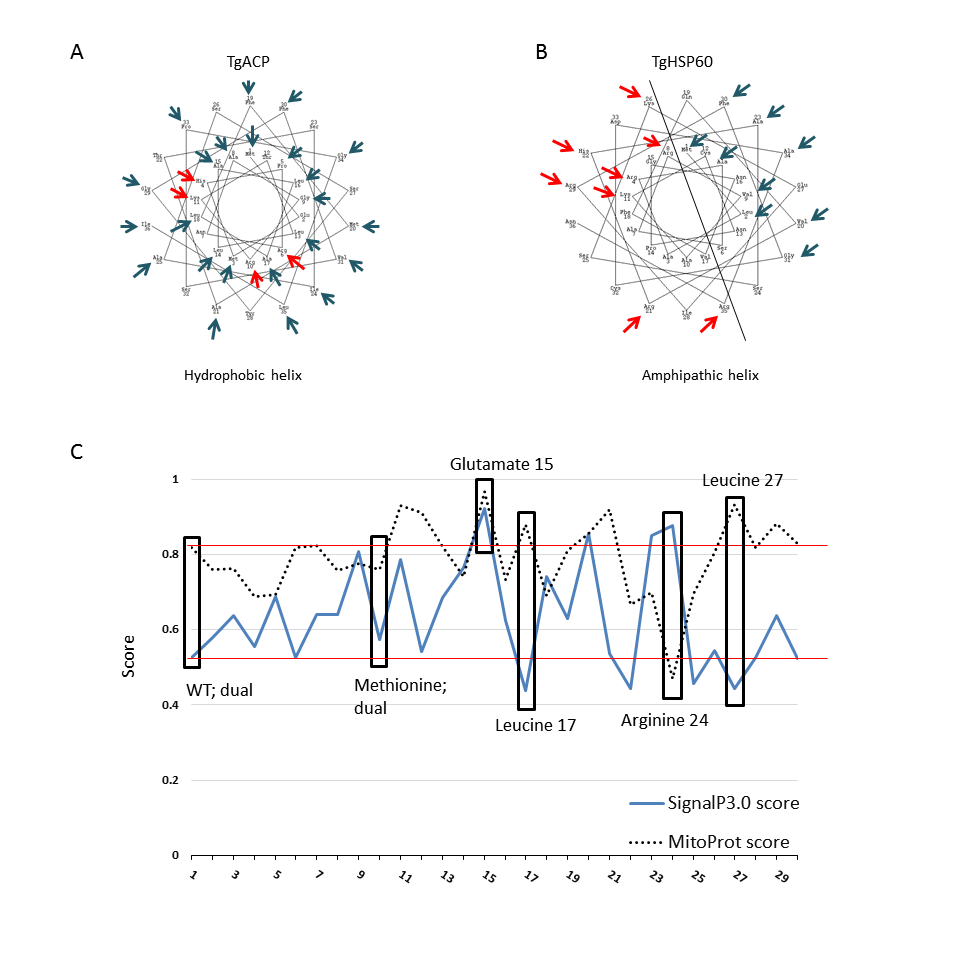


**Supplementary figure S3**: **Bioinformatics analysis of the N-terminus of apicoplast targeted TgACP and mitochondrial protein TgHSP60.** α-helix predictions for the N-terminus of A) apicoplast targeted protein TgACP and B) mitochondrial protein HSP60. The blue arrows mark the hydrophobic residues while the red arrows label the positively charged amino acids on the helical projections for the N-terminus of the proteins used for analysis.
